# Prevalence and patterns of antimicrobial resistance in *Escherichia coli* from Indonesian broiler chickens: Comparison of CLSI and EUCAST 2025 standards

**DOI:** 10.5455/javar.2026.m1050

**Published:** 2026-06-22

**Authors:** Ari Retnowati, Sugiharto Sugiharto, Dian Wahyu Harjanti

**Affiliations:** 1 National Laboratory of Veterinary Public Health, Ministry of Agriculture of the Republic of Indonesia, Bogor, Indonesia; 2 Faculty of Animal and Agricultural Sciences Diponegoro University, Semarang, Indonesia; 3 Postgraduate program of Animal Sciences, Faculty of Animal and Agricultural Sciences Diponegoro University, Semarang, Indonesia

**Keywords:** Broiler chicken, *Escherichia coli*, CLSI, EUCAST, antimicrobial resistance, antimicrobial surveillance

## Abstract

**Objectives:** This study aimed to compare the prevalence and resistance patterns of antimicrobial resistance (AMR) in *Escherichia coli* isolated from broiler chickens across Indonesia using the Clinical and Laboratory Standards Institute (CLSI) M100, 35^th^ edition (2025), and the European Committee on Antimicrobial Susceptibility Testing (EUCAST) version 15.0 (2025) guidelines.

**Material and Methods:** A total of 390 *E. coli* isolates were subjected to antimicrobial susceptibility testing using the broth microdilution method with the Sensititre EUVSEC3 panel. Resistance data were analyzed using the Chi-square test, Cohen’s kappa coefficient, and percent agreement.

**Results:** The highest resistance rates under both standards were observed for ampicillin (78.33% according to CLSI and 78.63% according to EUCAST), while all isolates remained susceptible to meropenem (100%) based on both references. Significant differences between CLSI and EUCAST interpretations were observed for colistin and ciprofloxacin (*p* < 0.05). Colistin demonstrated negligible agreement between the two standards (*κ* = –0.01; 95% CI: –0.05 to 0.03; 3% agreement). In contrast, interpretation of susceptibility results for ampicillin, cefotaxime, ceftazidime, gentamicin, and trimethoprim showed almost perfect concordance between CLSI and EUCAST (*κ* > 0.91).

**Conclusions:** Variations in the interpretation of antimicrobial susceptibility testing results may influence AMR surveillance outcomes and antimicrobial use decision-making in the field. Strengthening and harmonizing AMR surveillance reporting based on both standards is essential to support evidence-based and effective AMR mitigation policies. This approach, within a One Health framework, is to safeguard animal health, public health, and food safety in Indonesia.

## 1. Introduction

Antibiotic resistance/antimicrobial resistance (AMR) is currently one of the 10 global health threats, contributing to 1.27 million global deaths in 2019. The World Health Organization (WHO) stated in 2023 that AMR is classified as a silent pandemic because global deaths from it are estimated to reach 10 million per year by 2050 if effective prevention measures are not implemented. Antimicrobial resistance poses a significant threat to public health and global development [1].

One of the main factors driving the increase in AMR is the misuse of antibiotics in the livestock sector. Poultry can be a reservoir for the spread of AMR from animals to humans through the consumption of poultry products contaminated with resistant bacteria [2]. The presence of *Escherichia coli* in poultry that can produce Extended Spectrum β-Lactamase (ESBL) is capable of hydrolyzing β-lactam antibiotics, including third-generation cephalosporins: cefotaxime and ceftazidime [2, 3]. Therefore, controlling AMR requires a One Health approach that involves humans, animals, and the environment [4].

FAO data from 2020 confirms that the poultry sector plays a dominant role in the global food system. Chicken (poultry) production accounts for 40% of global meat production overall [5]. Broiler chicken farming in Indonesia is a leading sector for the national supply of animal protein. Indonesia is one of the countries with a relatively high consumption rate of chicken meat, reaching 7.4 kg per capita in 2024 [6]. Field practice shows that 40% of chicken farmers in Indonesia still use antibiotics for prevention rather than treatment of diseases [7]. Scientific knowledge and research on AMR in developing countries are still very limited [8]. As many as 55% of farmers in Indonesia do not have adequate understanding of AMR, so the inappropriate use of antibiotics is still frequently observed. This condition has the potential to increase the risk of antimicrobial resistance in the food chain. The prevalence of AMR in asymptomatic broiler chickens in Indonesia was reported to be 24.9% [9]. Additionally, non-clinical isolates from broiler chickens in several European countries (UK, Germany, and France) showed higher resistance to ampicillin and tetracycline compared to clinical isolates [10].

Antimicrobial susceptibility testing (AST) is crucial in AMR surveillance and selecting effective therapies for clinical treatment [11]. Indonesia has conducted AMR surveillance in the broiler chicken sector, but national surveillance-based data related to antibiotic resistance patterns on a national scale are still limited [12]. Globally, the guidelines used in interpreting AST testing are the Clinical Laboratory Standard Institute (CLSI) and the European Committee on Antimicrobial Susceptibility Testing (EUCAST). WHO recommends these two guidelines in the AMR surveillance system, also known as the Global Antimicrobial Resistance and Use Surveillance System (GLASS). The selection of guidelines for interpreting AST is crucial because of the increasing global focus on standardizing AMR data [13].

The CLSI standard is extensively used in Asian and American countries and most countries outside of Europe [14]. Meanwhile, the EUCAST standard, which is freely accessible, is periodically dominant in European countries such as France, Germany, Norway, Sweden, and the United Kingdom [15, 16]. There are important differences between these two references [17]. The breakpoints set by EUCAST are generally stricter than those of CLSI. The interpretation of breakpoints also differs between CLSI and EUCAST. Based on CLSI, interpretation categories include resistant (R), intermediate (I), and susceptible (S), whereas EUCAST only uses resistant and susceptible interpretations [7, 15, 18]. Differences in interpretation can affect the prevalence trends of AMR and can hinder data harmonization within the GLASS system, thus impacting antibiotic use policies [11, 13].

This study aims to assess the prevalence of AMR *E. coli* in broiler chickens in Indonesia using the 2024 national surveillance data and to compare interpretations based on CLSI 2025 and EUCAST 2025 guidelines. The findings are expected to provide a scientific basis for harmonizing AMR data and to support the development of policies for AMR control and surveillance in Indonesia, in alignment with both national and global priorities.

## 2. Material and Methods

### 2.1. Ethical statement

The investigation was carried out according to the animal ethics committee of Diponegoro University. Ethical approval number: 62-04/A-09/KEP-FPP, issued by the Animal Ethics Committee at Diponegoro University.

### 2.2. Sampling design

**Figure 1.**
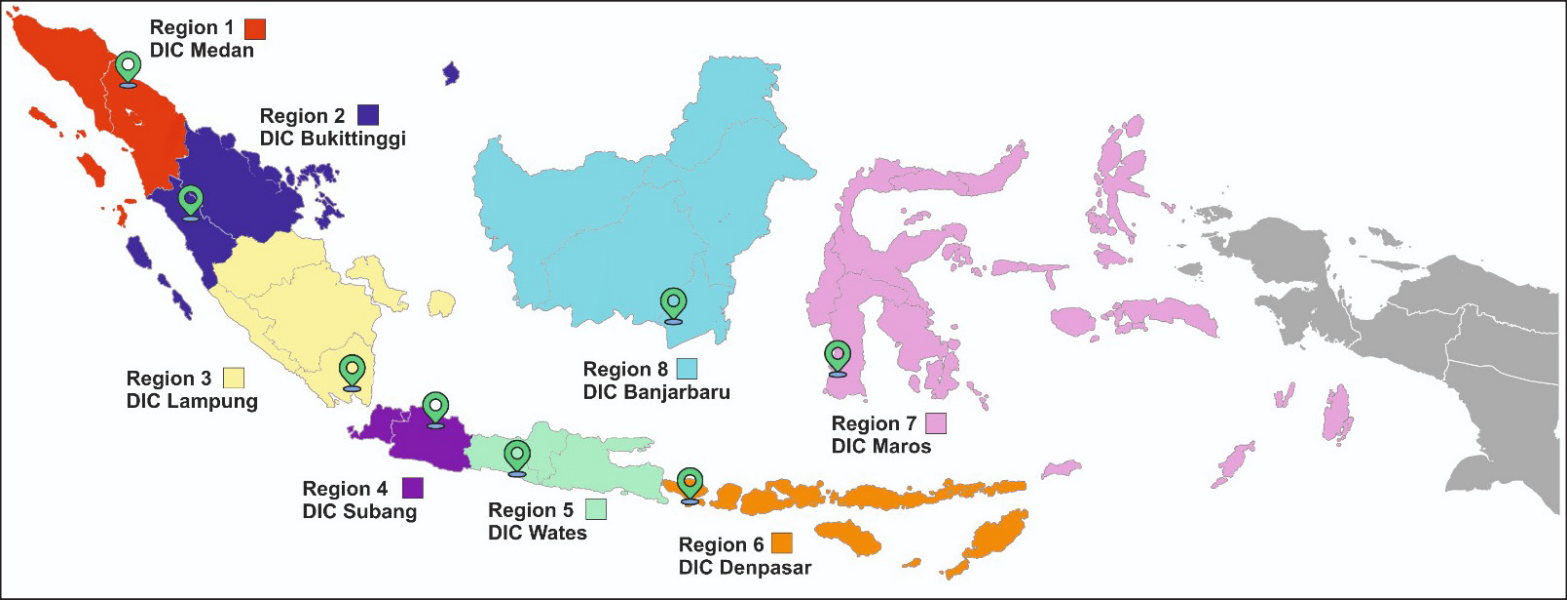
Distribution of AMR surveillance samples spread in 2024 (DIC: Disease Investigation Center).

These isolates were obtained through the National AMR Surveillance program in 2024, originally collected and isolated by eight Disease Investigation Center (DIC) regions across Indonesia (Figure 1), following the National AMR Surveillance Guidelines issued by the Directorate of Veterinary Public Health, Ministry of Agriculture [19]. Sampling was conducted at the Poultry Slaughterhouse (RPH-U), Poultry Slaughtering Facility (TPU), or Poultry Holding Facility (TPNU). Samples were taken during the slaughtering process at each sampling unit. One healthy broiler chicken was randomly collected from each broiler chicken originating from different farms to avoid cluster bias. The sampling process was repeated every two weeks.

### 2.3. E. coli isolation

Isolation and identification of *E. coli* were carried out at the animal health laboratory of eight DICs. Cecal samples were inoculated onto MacConkey agar and incubated at 35–37°C for 18–24 h. Separated *E. coli* colonies (pink in color) were inoculated onto nutrient agar and subjected to biochemical confirmation using indole, methyl red, voges-proskauer, and citrate (IMViC) tests. Confirmed isolates were preserved in tryptic soy broth (TSB) supplemented with 20% glycerol and stored at –20°C. All isolates were subsequently transported under cold chain conditions to the National Veterinary Public Health Laboratory (BPMSPH) in Bogor for centralized curation and susceptibility testing. The number of isolates (*n* = 390) per region varied between 43 and 50 isolates (Table 1).

**Table 1.** *Escherichia coli* isolates surveillance AMR in 2024.

DIC Regions	District	Number
1. Medan	North Sumatra, Aceh	50
2. Bukittinggi	West Sumatera, Riau, Jambi, Riau Island	49
3. Lampung	Lampung, South Sumatra, Bengkulu, Bangka Belitung	49
4. Subang	West Java, Jakarta	50
5. Wates	Central Java, East Java, Yogyakarta	50
6. Denpasar	Denpasar, West Nusa Tenggara, East Nusa Tenggara	50
7. Maros	Gorontalo, Maluku, Sulawesi	49
8. Banjarbaru	Banjarbaru, East Kalimantan	43
	Total	390

DIC: Disease Investigation Center.

### 2.4. Antimicrobial susceptibility testing

All 390 isolates were subjected to antimicrobial susceptibility testing (AST) against a panel of 15 antibiotics and compared using different standards (CLSI and EUCAST). Antimicrobial susceptibility testing was done using the broth microdilution method following the guidelines in CLSI M100, 35^th^ edition, 2025 [14]. Isolates were tested using Sensititre EUVSEC3 microplates (Thermo Fisher Scientific, USA) with 15 types of antibiotics with concentration ranges: Amikacin (AMK) 4–128 µg/ml; Ampicillin (AMP) 1–32 µg/ml; Azithromycin (AZI) 2–64 µg/ml; Cefotaxime (FOT) 0.25–4 µg/ml; Ceftazidime (TAZ) 0.25–8 µg/ml; Chloramphenicol (CHL) 8–64 µg/ml; Ciprofloxacin (CIP) 0.015–8 µg/ml; Colistin (COL) 1–16 µg/ml; Gentamicin (GEN) 0.5–16 µg/ml; Meropenem (MER) 0.03–16 µg/ml; Nalidixic Acid (NAL) 4–64 µg/ml; Sulfamethoxazole (SMX) 8–512 µg/ml; Tetracycline (TET) 2–32 µg/ml; Tigecycline (TGC) 0.25–8 µg/ml; Trimethoprim (TMP) 0.25–16 µg/ml [20].

Minimum Inhibitory Concentration (MIC) values were recorded using a Sensititre plate reader and SWIN software system (Thermofisher Scientific, USA). MIC results were interpreted using reference breakpoints based on CLSI M100 35th Edition, 2025, and EUCAST v15.2025. Interpretations were categorized as Resistant (R) – Intermediate (I) – Susceptible (S) for CLSI 2025 and R-S for EUCAST 2025 [14, 15, 21]. Antibiotics NAL, TET, AZI, CHL, and SMX were interpreted only using CLSI M100 2025, because EUCAST 2025 does not establish breakpoints for these antibiotics against *E. coli* isolates [15]. Data were compiled and summarized using Microsoft Excel 2013.

### 2.5. Statistical analysis

For statistical analysis purposes, the AST interpretation data were categorized dichotomously into two groups: non-susceptible (R + I) and susceptible (S). The inclusion of category I in category R was based on considerations for AMR surveillance. The merging of category I isolates in the CLSI 2025 standards into the ‘NS’ group serves as a conservative early warning approach, prioritizing the detection of emerging resistance in AMR surveillance. In addition, since 2019, the EUCAST has redefined the I category as “susceptible increased exposure”. This study categorized it as NS to maintain a high sensitivity threshold for surveillance purposes. Dividing categories into NS and S groups ensured a uniform binary scale for the agreement analysis between two standards. However, it is recognized that this dichotomization may impact the categorical agreement between the two standards, potentially leading to lower agreement in isolates where the I category is frequent.

Differences in resistance proportions between CLSI 2025 and EUCAST 2025 interpretations were analyzed using Pearson’s chi-square test (χ²). A χ²*p* value < 0.05 was considered a statistically significant difference between the two references. The level of agreement between the two references was analyzed using Cohen’s Kappa with a 95% confidence interval (95% CI) and the percentage of agreement. The interpretation of Cohen’s Kappa ranges from poor agreement (*κ* < 0) to almost perfect agreement (*κ* = 0.81–1.00) [22].

Statistical analyses were only performed for 10 antibiotics (Table 3). Five antibiotics (AZI, CHL, NAL, SMX, and TET) were excluded from statistical comparative analysis because EUCAST 2025 does not establish breakpoints for these antibiotics against *E. coli*, so interpretation was based solely on CLSI 2025. All statistical analyses were performed using R software (RStudio version 4.5.2) with a significance level of *p* < 0.05.

## 3. Results

**Figure 2.**
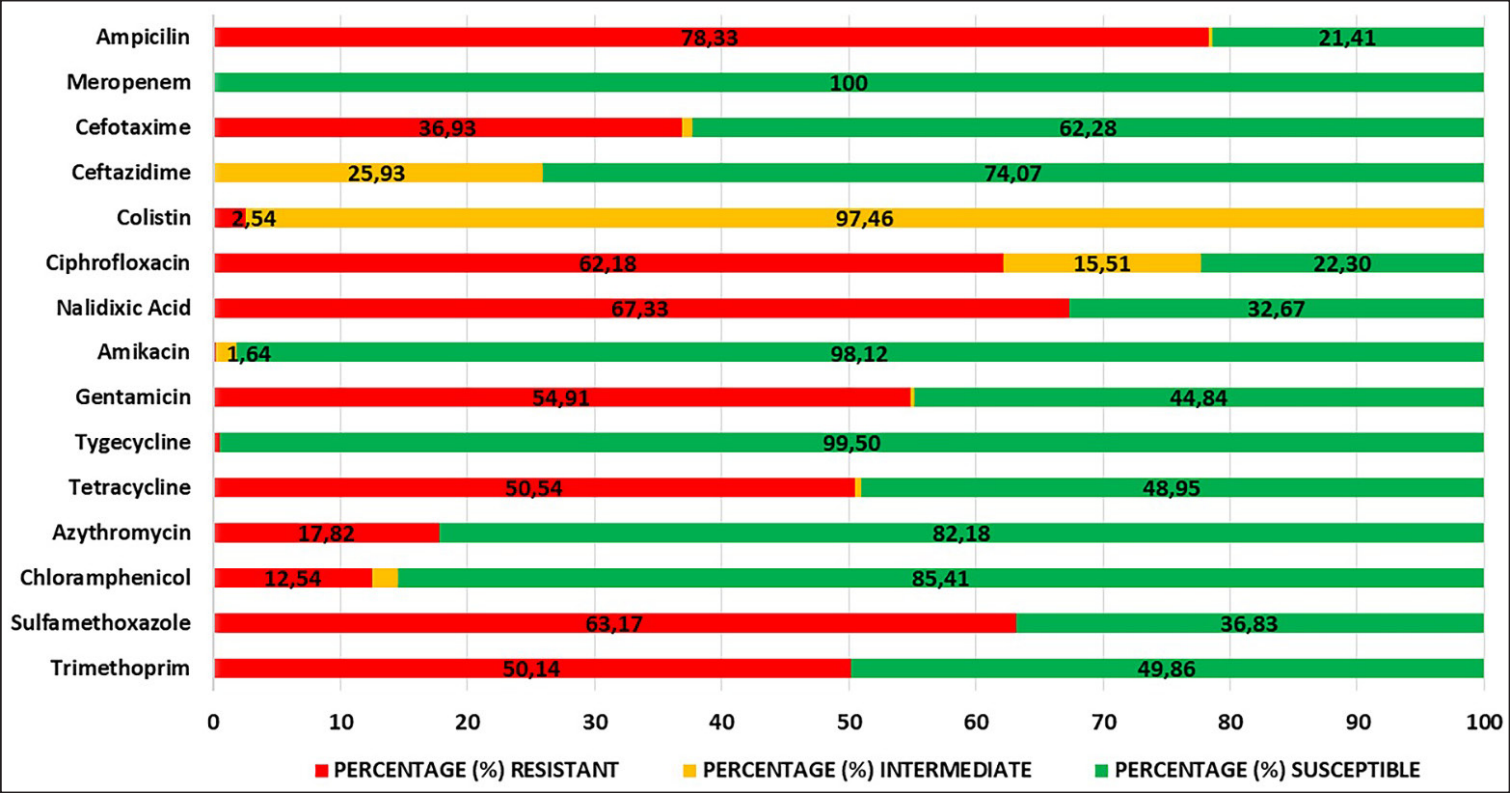
Antimicrobial susceptibility resistance patterns based on CLSI 2025.

**Figure 3.**
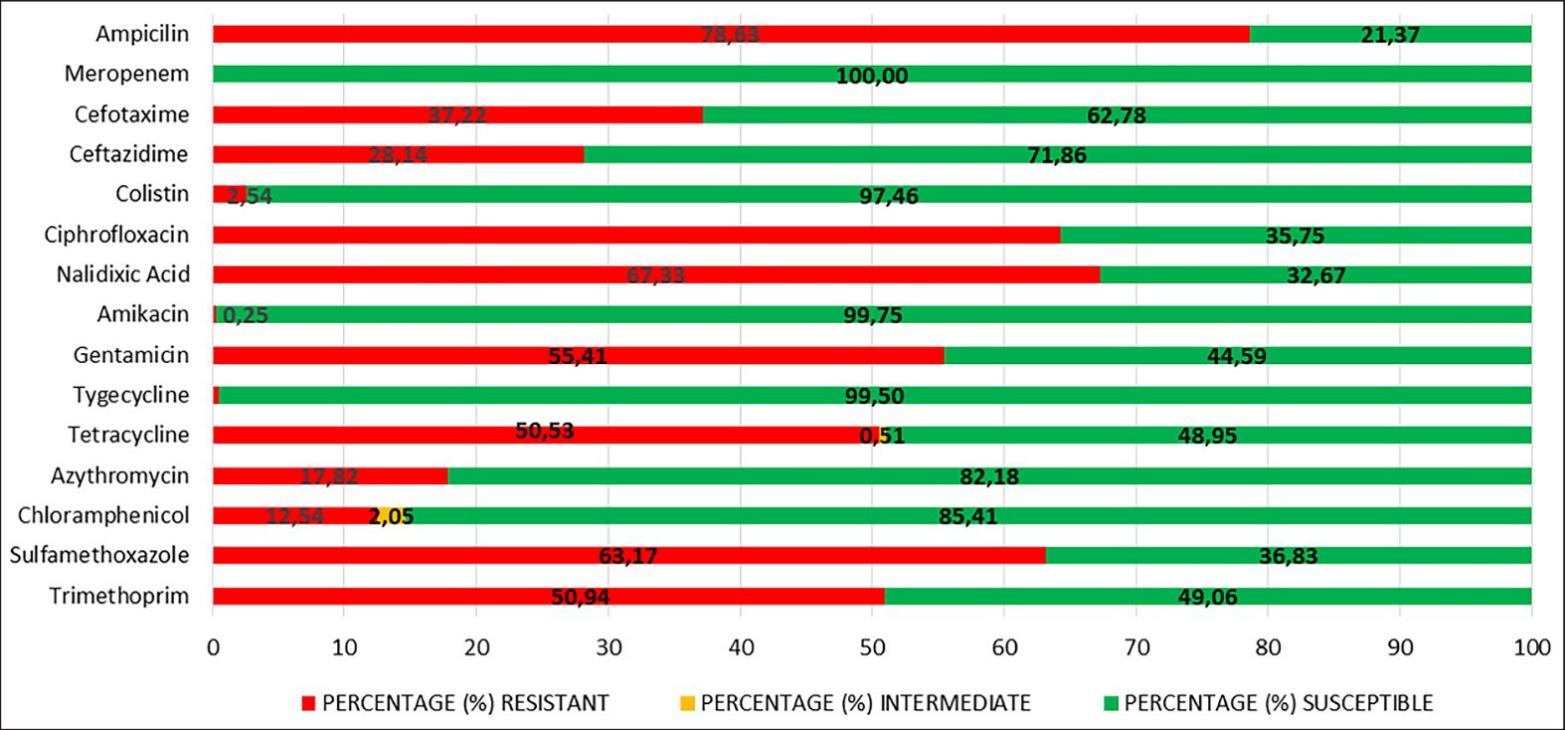
Antimicrobial susceptibility resistance patterns based on EUCAST 2025.

The visualization results of the antimicrobial susceptibility test resistance pattern interpretation based on CLSI 2025 and EUCAST 2025 are shown in Figures 2 and 3. Resistance prevalence data were recapitulated and processed using Microsoft Excel 2013 and summarized in Table 2. Overall, both references showed diverse antibiotic resistance patterns. CLSI 2025 utilizes the Resistant-Intermediate-Susceptible (R-I-S) categories, while EUCAST 2025 uses the Resistant-Susceptible (R-S) categories.

**Table 2.** Resistance patterns based on antimicrobial susceptibility categories according to CLSI 2025 and EUCAST 2025.

Antibiotic	Class**	CLSI 2025 (∑ = 390)			EUCAST 2025 (∑ = 390)		
		R (n)	I (*n*)	S (*n*)	R (*n*)	I (*n*)	S (*n*)
Ampicillin	Penicilin	305	1	84	306	–	84
Meropenem	Carbapenem	0	0	390	0	–	390
Cefotaxime	Cephalosphorine	145	3	242	146	–	244
Ceftazidime	0	102	288	110	–	280
Colistin	Polymixin	10	380	0	10	–	380
Ciprofloxacin	Quinolon	242	60	88	250	–	140
Nalidixic Acid	262	–	128	262*	–	128*
Amikacin	Aminoglycoside	1	6	383	1	–	389
Gentamicin		214	1	175	216	–	174
Tigecycline	Tetracycline	2	–	388	2	–	388
Tetracycline		197	2	191	197*	2*	191*
Azithromycin	Macrolide	70	–	320	70*	–‘	320*
Chloramphenicol	Chlorampenicol	49	8	333	49*	8*	333*
Sulfamethoxazole	Sulfonamide	247	–	143	247*	–	143*
Trimethoprim		196	–	194	199	–	191

*: interpretation using CLSI 2025 reference; R, resistant; I, intermediate; S, susceptible. **Antibiotic classification based on WHO [23].

**Table 3.** Statistical analysis results of antimicrobial susceptibility tests between CLSI 2025 and EUCAST 2025.

Antibiotic	CLSI 2025 (*n* = 390)			EUCAST 2025 (*n* = 390)		χ^2^ *p*-Value	Kappa (95% CI)	Notes	% Agreement
	NS (%)		S (%)	NS (%)	S (%)				
	R	I	S	R	S				
Ampicillin	78.33	0.26	21.41	78.63	21.37	1.00	0.97 (0.92; 1.00)	Almost perfect	100
Meropenem	0	0	100	0	100	n.p.	n.p.	n.p.	n.p.
Cefotaxime	36.93	0.79	62.28	37.22	62.78	0.88	0.96 (0.90; 1.00)	Almost perfect	99
Ceftazidime	0	25.93	74.07	28.14	71.86	0.75	0.93 (0.84; 1.00)	Almost perfect	98
Colistin	2.54	97.46	0	2.54	97.46	<0.001^a^	–0.01 (–0.05; 0.03)	Poor	3
Ciprofloxacin	62.18	15.51	22.31	64.25	35.75	0.03^b^	0.65 (0.49; 0.82)	Substantial	86
Amikacin	0.25	1.64	98.12	0.25	99.75	0.16	0.24 (–0.61; 1.00)	Fair	98
Gentamicin	54.91	0.25	44.84	55.41	44.59	1.00	0.98 (0.94; 1.00)	Almost perfect	100
Tigecycline	0.50	–	99.50	0.50	99.50	1.00	0.75 (0.25; 1.00)	Substantial	100
Trimethoprim	50.14	–	49.86	50.94	49.06	0.89	0.96 (0.91; 1.00)	Almost perfect	99

NS, non-susceptible; n.p., not performed; ^a,b,^ significant; R, resistant; I, intermediate; S, susceptible.

Both references identified ampicilin as having the highest resistance rate. The prevalence of resistance rate according to CLSI 2025 was 78.33% (305/390) and 78.63% (306/390) according to EUCAST 2025. Furthermore, resistance of over 50% was also observed for trimethoprim, gentamicin, and ciprofloxacin for both references, with EUCAST 2025 having a slightly higher resistance proportion than CLSI 2025. All isolates were susceptible (100%) to meropenem according to both references. Interpretation of cefotaxime showed a consistent resistance pattern between both references, although there was an increase in proportion in EUCAST 2025. Based on CLSI 2025, 36.93% (145/390) of isolates were categorized as resistant to cefotaxime, and 37.22% (146/390) based on EUCAST 2025.

The interpretation of ceftazidime, ciprofloxacin, and colistin showed numerical differences between the two references. According to CLSI 2025, 25.93% (102/390) of isolates were categorized as intermediate to ceftazidime, while 28.14% (110/390) were categorized as resistant under EUCAST 2025. CLSI 2025 categorized 62.18% (242/390) of isolates as resistant to ciprofloxacin and 22.31% (88/390) as susceptible. Meanwhile, EUCAST 2025 showed higher resistance and susceptible rates. Colistin showed major numerical differences that 97.46% (380/390) were interpreted as intermediate according to CLSI 2025 (which does not provide a susceptible breakpoint for this antibiotic), while based on EUCAST 2025, these isolates were interpreted as susceptible. Interpretation for amikacin, gentamicin, and trimethoprim remained consistent between both references, although EUCAST 2025 had slightly higher values than CLSI 2025. Across both references, tigecycline showed high susceptibility at 99.50% (388/390).

Statistical analyses, chi-square, Cohen’s Kappa, and percentage agreement could not be calculated for meropenem (not performed/n.p.). It had 100% susceptibility under both references, leading to no categorical variation. Statistical analysis (Table 3) confirmed that significant differences between the two references existed only for colistin (*p* < 0.001) and ciprofloxacin (*p* = 0.03). Meanwhile, ampicillin, cefotaxime, ceftazidime, amikacin, gentamicin, tigecycline, and trimethoprim showed no statistically significant differences in interpretation (*p* > 0.05) between the two references.

Cohen’s kappa and percent agreement analysis (Table 3) demonstrated that the level of agreement between CLSI 2025 and EUCAST 2025 varied across different antibiotics. Almost perfect agreement levels were shown for ceftazidime (*k* = 0.93; CI:0.84–1; 98%), trimethoprim (*k* = 0.96; CI:0.91–1; 99%), cefotaxime (*k* = 0.96; CI:0.90–1; 99%), ampicilin (*k* = 0.97; CI:0.91–1; 100%), and gentamicin (*k* = 0.98; CI:0.94–1; 100%). Substantial agreement was shown for tigecycline (*k* = 0.75; CI: 0.25–1; 100%) and ciprofloxacin (*k* = 0.65; CI: 0.49–1; 86%) between the two references. Fair agreement between the two references was observed for amikacin (*k* = 0.24; CI: –0.61–1; 98%). In contrast, colistin was observed to have poor agreement (*k* = –0.01; CI: –0.05–0.03; 3%).

## 4. Discussion

The effectiveness of antimicrobials in treating bacterial infections is a critical issue for both the animal and human health sectors [24]. Therefore, the results of the antimicrobial susceptibility test (AST) play an important role in determining therapy for bacterial infections. Reporting AST results is a strategic tool for antimicrobial resistance (AMR) control, supporting clinical decision-making, empirical therapy selection, and the formulation of antibiotic use policies as part of AMR mitigation efforts, including in the poultry sector [8, 25].

This study revealed high resistance levels to ampicillin (78%), ciprofloxacin (62–64%), gentamicin (54–55%), and trimethoprim (50%) according to CLSI 2025 and EUCAST 2025 references. The lowest resistance was found for amikacin (0.25%). In contrast, the AMR surveillance study in Indonesia between 2018 and 2020 revealed very high rates of resistance to ampicillin (97.5%) and cefotaxime (98.5%), with the lowest rates of resistance to tigecycline (2.5%) [26]. Studies from various regions, including Malawi (East Africa), Algeria (North Africa), and Bangladesh, reported remarkably high tetracycline resistance in broiler chickens (90%, 100%, and 100%, respectively) [27, 28, 29]. Globally, tetracycline usage is projected to increase by 8% by 2030, which could create selective pressure and exacerbate the prevalence of antimicrobial resistance [30]. Tetracycline resistance was still detected in this study at a rate of 50% according to both references (Figures 2, 3), lower than in other countries. Our study showed that ampicillin is the primary concern in Indonesia, even though tetracycline is the most common resistance profile globally.

Ampicilin and cefotaxime are β-lactam antibiotics that work by inhibiting the synthesis of bacterial cell walls [31]. In poultry farming, ampicillin is frequently used as the first-line therapeutic antibiotic. Globally, ampicilin was frequently used for treating *E. coli* and *Salmonella* infections due to its wide spectrum and also affordable cost [32]. Research on broiler farms in Malaysia, China, and Algeria reported resistance rates to ampicillin reaching 76%, 95%, and 97% [33, 34, 35]. Meanwhile, Korea showed high resistance to β-lactams at 84% [36]. These findings indicated that β-lactam antibiotics were frequently used on broiler farms, contributing to the observed high resistance levels. Antimicrobial use (AMU) in livestock had been identified as one of the contributors to the emergence of antimicrobial resistance in bacteria, impacting animal, environmental, and human health [37].

Amid concerns regarding antimicrobial resistance, the WHO recommends restricting the use of cefotaxime in food-producing animals to suppress the emergence of resistance that impacts human health. Cefotaxime is considered a Highest Priority Critically Important Antimicrobial (HPCIA) in the treatment of severe infections in humans [38]. This study showed a resistance rate of 36–37% to cefotaxime based on both references, which was a decrease compared to the 2018–2020 AMR surveillance period, where it reached 98.5%. The Ministry of Agriculture has banned the use of critical antibiotics, including colistin, ciprofloxacin, and third-generation cephalosporins orally, parenterally, and topically in food-producing animals [39]. This policy can be a strategic mitigation step to break the chain of AMR spread in the food-producing livestock sector. Resistance to that class of antibiotics can reduce the effectiveness of therapy in humans. Surveillance AMR data 2024 showed that resistance to ciprofloxacin and cefotaxime remains quite high based on CLSI and EUCAST 2025 references. These findings might indicate a widespread presence of resistance to quinolone and third-generation cephalosporin antibiotics in the field. While these results underscore the ongoing challenges in controlling AMR, further studies incorporating AMU data are needed to accurately assess the long-term efficacy of current government restriction policies.

In contrast, all *E. coli* isolates (100%) from the 2024 AMR surveillance were identified as susceptible to meropenem according to both references. This finding was slightly higher than the results of the 2018–2020 surveillance period, which reported a susceptibility rate of 96.5% [26]. The results indicated that resistance, specifically meropenem (carbapenem) resistance in *E. coli* from broiler chickens in Indonesia, was still undetected or extremely low. This condition aligns with global surveillance reports, which show a very low prevalence of carbapenem resistance in the poultry sector [28, 40].

According to the CLSI 2025 reference, colistin susceptibility testing showed that 97.46% of isolates were categorized as Intermediate (I) and 0% as Susceptible (S). In contrast, according to EUCAST 2025, 97.46% of isolates are susceptible. When R + I categories were grouped into Non-Susceptible (NS) for statistical analysis, it resulted in a highly significant difference (*p* < 0.001) between the two references. Both references showed a systematic lack of agreement, as seen in the negative kappa value (*k* = –0.01; CI: –0.05–0.03; 3%). This discrepancy is due to fundamental differences in breakpoint values between CLSI 2025 and EUCAST 2025.

CLSI reclassified the S category to the I category (MIC ≤ 2 µg/ml) in 2020 due to concerns regarding the limitations of laboratory testing methods and the efficacy of clinical therapy. Conversely, EUCAST 2025 maintains the S category for the same range (MIC ≤ 2 µg/ml) based on the Epidemiological Cut-Off Values (ECOFFs) approach to its interpretation, assuming that wild-type isolates could still be considered susceptible to colistin [41]. Different interpretations in colistin between CLSI 2025 and EUCAST 2025 could lead to inconsistencies in isolate classification in global surveillance data reporting, as evidenced by the findings of this study. Negative kappa in this finding indicated that the disagreement between the two references is not random but a direct result of their opposing categorical definitions.

Statistical analysis additionally revealed a significant difference (*p* = 0.03) in ciprofloxacin interpretations between the two references despite a substantial level of agreement (*k* = 0.65; CI: 0.49–0.82; 86%). This discrepancy was primarily driven by the proportion of isolates categorized as I under CLSI 2025. Specifically, CLSI 2025 categorized 15.51% of isolates as I, 22.31% as S, and 62.18% as R, whereas EUCAST 2025 categorized 35.75% of isolates as S and 64.25% as R. By consolidating categories I and R into the NS group for statistical analysis comparison, the NS value in CLSI 2025 appeared higher than in EUCAST 2025. These discrepancies could potentially lead to conflicting therapeutic decisions in the field, thereby posing risks to clinical efficacy and antimicrobial stewardship.

In contrast, tigecycline showed very high susceptibility (99.5%) according to both references. Statistical analysis of tigecycline showed 100% agreement with substantial concordance (*k* = 0.75; CI: 0.25–1; 100%). This might be due to the very high prevalence difference between resistant and susceptible isolates, whose prevalence of the NS category between the two references was < 1%. These findings meant that CLSI 2025 and EUCAST 2025 references had consistent interpretations for tigecycline susceptibility.

Statistical analysis for amikacin revealed a fair level of agreement (*k* = 0.24; CI: –0.61 to 1; 98%), despite having a very high percent agreement (98%). This phenomenon is identified as the kappa paradox, where the kappa value remains low despite the high agreement value [42]. This phenomenon occurs due to uneven distribution of categories across the two references, CLSI 2025 and EUCAST 2025. The prevalence of amikacin in the S category was over 98% in both CLSI 2025 and EUCAST 2025, compared to the NS category, which was a minimal 1.89% in CLSI 2025 and 0.25% in EUCAST 2025. This research showed that both reference standards had consistent categories for S, although they exhibit inconsistent interpretations for the NS category. This was due to the difference in breakpoints between the two standards. Overall, the research results confirmed that resistance to amikacin and tigecycline in broiler chickens in Indonesia remains extremely low.

The difference in interpretation approaches between CLSI 2025 and EUCAST 2025 pertains to the intermediate category; unlike the CLSI 2025 reference, EUCAST 2025 redefined intermediate interpretations in 2019. The intermediate category in EUCAST 2025 indicates that an increased dose (increased exposure) of antimicrobials is required for successful clinical treatment. CLSI maintains the intermediate category as a technical buffer zone to accommodate variations in MIC results and prevent misclassification of isolates as resistant [14, 15, 21, 43]. The findings of this study are consistent with previous research indicating that differences in breakpoints between CLSI 2025 and EUCAST 2025 can lead to varied resistance profiles. EUCAST 2025 tends to report higher resistance percentages due to stricter breakpoint criteria. These interpretation differences have important clinical and epidemiological implications for the interpretation of AMR surveillance data [16, 43].

Furthermore, the interpretative discrepancies between CLSI 2025 and EUCAST 2025 have a significant impact on AMR control policies in the poultry sector, which indirectly contributes to environmental and public health risks. AMR surveillance data is used as the foundation for developing the National Action Plan (NAP). The reference interpretation based on EUCAST 2025, which shows higher resistance compared to CLSI 2025, can function as a robust early warning system. Adopting this conservative approach may help mitigate the risk of AMR spreading from the poultry sector, thereby safeguarding public health through the food chain.

## 5. Conclusions

*Escherichia coli* isolates from Indonesian broiler chickens exhibited high resistance to ampicillin but remained fully susceptible to meropenem under both CLSI and EUCAST 2025 standards. While most antimicrobials showed strong concordance between the two references, only colistin and ciprofloxacin exhibited significant discrepancies. These highlight the need for harmonized AMR reporting. Such variations in the interpretation of antimicrobial susceptibility testing results may directly influence AMR surveillance outcomes and antimicrobial use decision-making in the field. Therefore, strengthening and harmonizing AMR surveillance reporting based on both standards is essential to support evidence-based and effective AMR mitigation policymaking. Adopting this harmonized approach within a One Health framework is safeguarding animal health, public health, and food safety in Indonesia.

## Data Availability

The data presented in this study are available from the corresponding author upon reasonable request.

## List of abbreviations

AMK, amikacin; AMP, ampicillin; AMR, antimicrobial resistance; AMU, antimicrobial use; AZI, azithromycin; CHL, chloramphenicol; CIP, ciprofloxacin; CLSI, Clinical and Laboratory Standards Institute; COL, colistin; DIC, Disease Investigation Center; ECOFFs, Epidemiological Cut Off Values; EUCAST, European Committee on Antimicrobial Susceptibility Testing; FOT, cefotaxime; GEN, gentamicin; GLASS, Global Antimicrobial Resistance and Use Surveillance System; HPCIA, Highest Priority Critically Important Antimicrobial; I, intermediate; MIC, Minimum Inhibitory Concentration; MER, meropenem; NAL, nalidixic acid; n.p., not performed; NS, non-susceptible; R, resistance; S, susceptible; SMX, sulfamethoxazole; TAZ, ceftazidime; TET, tetracycline; TGC, tigecycline; TMP, trimethoprim; WHO, World Health Organization; χ², chi-square statistic; κ, Cohen’s kappa coefficient; kg, kilogram; µg/ml, microgram per milliliter.
